# Hybrid and Chimeric Heterocycles for the Inhibition of Carbonic Anhydrases

**DOI:** 10.3390/ph18091387

**Published:** 2025-09-16

**Authors:** Niccolò Paoletti, Simone Giovannuzzi, Claudiu T. Supuran

**Affiliations:** NEUROFARBA Department, Section of Pharmaceutical Science, University of Florence, Via Ugo Schiff 6, Sesto Fiorentino, 50019 Florence, Italy; niccolo.paoletti@unifi.it (N.P.); claudiu.supuran@unifi.it (C.T.S.)

**Keywords:** heterocycles, carbonic anhydrase inhibition, multitarget approach, chimeric inhibitor

## Abstract

The design of multitarget drugs is a growing strategy to address complex and multifactorial diseases, and heterocycles play a major role in this approach. This review aims to critically analyze the role of heterocyclic scaffolds in the development of human carbonic anhydrase inhibitors (hCAIs), emphasizing their versatility as core chemotypes, linkers, and secondary pharmacophores. By examining advances from the last 10 years, we highlight how heterocycle-based designs contribute to modulating potency and selectivity toward hCAs, as well as to the creation of hybrid molecules with enhanced therapeutic profiles. Understanding these strategies is essential for guiding future drug discovery efforts targeting hCAs and related pathologies.

## 1. Introduction

Heterocycles constitute a broad class of molecules found in many biologically active compounds in the human body [1]. Their natural abundance, structural complexity, and ability to form hydrogen bonds make heterocycles particularly attractive for pharmaceutical applications [2]. Particularly, their use falls under a wide set of applications in multitargeting strategies by adopting both heterocycles and/or chimeric moieties [3,4,5]. Briefly, a multitarget strategy involves designing drugs that can interact with multiple biological targets to treat complex diseases [3,6,7]. This approach is an alternative to traditional “one drug, one target” therapies [6], offering potential advantages in efficacy and safety for multifactorial conditions [7]. This strategy involves combining pharmacophores from different bioactive molecules into a single chemical entity [3,4,5,6,7]. Among the various applications, this can be achieved either by linking two distinct pharmacophores with an appropriate spacer or linker or by merging the active pharmacophores into a single structural framework, consequently generating a unified scaffold (chimeric scaffold) [7]. These compounds have garnered significant interest due to their capacity to interact with multiple enzymes and modulate their activity.

The study of heterocycles as inhibitors of human carbonic anhydrase (hCA) activity has emerged as a promising strategy for therapeutic intervention [8,9]. hCAs constitute a family of drug targets characterized by multiple isoforms with distinct cellular localizations and physiological roles, which can vary significantly across tissues and pathological conditions [10]. The 12 catalytically active isoforms exhibit a central role in processes such as pH homeostasis, respiration, and CO_2_/HCO_3_^−^ transport [10]. They also contribute to key biosynthetic pathways, including gluconeogenesis, lipogenesis, and ureagenesis, all of which are essential for cellular metabolism [11,12,13]. Dysregulation of hCAs has been linked to a wide spectrum of disorders, including glaucoma, inflammatory conditions, hypoxic tumors, and neurodegenerative diseases [14]. As a consequence, over time, the inhibition of hCA isoforms has become an important therapeutic approach, offering promising avenues for the treatment of diverse diseases [11,12,13,14].

Despite the large number of reviews published in the carbonic anhydrase field in recent years [10,11,12,13,14,15,16,17,18,19,20], this review summarizes, for the first time, the role of hybrid and chimeric heterocycles in carbonic anhydrase inhibition produced in the last 10 years, focusing on their use as scaffolds, linkers, and multitarget-directed moieties to enhance CA binding.

## 2. Heterocycles Used as Carbonic Anhydrase Inhibitory Chemotype

Heterocycles as CA inhibitory chemotypes have been extensively used in the last 5–10 years. Particularly, coumarins represent the most commonly adopted scaffold in this field, with the aim of selectively targeting the hCAs IX and XII, mostly involved in the pathogenesis of hypoxic cancer and inflammation. However, as will be discussed in this section, several other heterocycles have been adopted in multitargeting compounds to address the inhibition of CAs, such as sulfocoumarins, pyridylsulfonamides, and tetrazoles.

### 2.1. (Sulfo)coumarins Are Potent Carbonic Anhydrase Inhibitors

One of the first hybrid heterocycle series against CAs was published by Bua et al. in 2017 [21], in which they designed and synthesized a series of small molecular hybrids to treat rheumatoid arthritis (RA), a chronic and systemic inflammatory disease caused by a faulty autoimmune response. The series contained a selective hCA IX and XII inhibitor head linked through a physiological cleavable linker to a carboxylic acid NSAID (COX inhibitor) tail in a 1:1 ratio [21]. For the NSAIDs, indomethacin, sulindac, ketoprofen, ibuprofen, diclofenac, flurbiprofen, ketorolac, and naproxen were selected. All derivatives were tested for their inhibitory activity against a panel of hCAs, that are isoforms I, II, IV, IX, and XII, by a kinetic assay, and the most promising candidates, **1**–**5**, are reported in Table 1. Compounds **1** and **2** resulted in selective inhibitors of the membrane-associated CAs, with K_I_ values ranging in the low-to-medium nanomolar range. Conversely, derivatives **4** and **5** exhibited selectivity for hCA IV, with K_I_ values in the low nanomolar range. These hybrids, **1**–**5**, were further assessed in vivo for their pain relief efficacy in a rat model of RA induced by intra-articular complete Freund’s adjuvant injection. Among them, compounds **1**, **4**, and **5** induced a significant reduction in hypersensitivity, showing comparable efficacy with the reference drug ibuprofen [21].

A few years later, in 2020, the same research group proposed a bioisosteric development of the previous series [22]. Indeed, an additional set of hybrid compounds containing an NSAID and a coumarin fragment was designed by substituting the amide moiety with an ester, which more easily undergoes cleavage caused by esterases and/or acidic conditions present in the target tissues. Such cleavable derivatives were assayed for their inhibition against CAs of interest, and the best performing are reported in Table 1, **6**–**10**. Such derivatives exhibited an interesting profile against isoforms IV, IX, and XII, showing low-to-medium nanomolar values [22]. Among them, derivative **8** showcased a single-digit nanomolar K_I_ value against hCA IX, making it the most potent inhibitor of this series. Furthermore, plasma stability studies showed the achievement of prodrug compounds because derivatives **6**–**10** reported a quick cleavage in rat plasma, and **6** and **9** were also subjected to human plasma esterases [22]. They were further assessed in vitro for their activity against COX-1 and -2, demonstrating that ketoprofen ester derivatives **6** and **9** promoted a small decrease in the COX-1-related anti-inflammatory effect, maintaining a comparable PgE2 level. Consequently, with derivatives **6** and **9** being the most promising candidates, they were further in vivo evaluated by the paw-pressure and incapacitance tests using an RA model. Of note, **6** exhibited major efficacy compared with coadministration in equimolar doses of its single pharmacophores [22].

In the same year, Berrino et al. proposed a novel series of coumarins and sulfocoumarins linked to azidothymidine (AZT), the first antiretroviral medication used to prevent and treat HIV/AIDS [23]. AZT was recently repurposed for its ability to bind preferentially to telomeres, inhibit telomerase, and enhance tumor cell senescence and apoptosis in breast mammary adenocarcinoma cells [24]. Based on this prompt, the authors designed and synthesized a series of CAIs bearing AZT with the aim of treating cancer, targeting two crucial players in cancer progression, telomerase and CAs. The set of (sulfo)coumarins includes seven new compounds, **11**–**17**, with remarkable activity against the cancer-associated hCA XII (Table 2) [23]. Among them, the sulfocoumarin-based inhibitor, **17**, exhibited the most potent activity against hCA XII, with a K_I_ of 2.8 nM, making it at least 2-fold more effective than its congeners and the standard drug **AAZ**. On the other hand, derivatives **11**–**17** did not show potent inhibition of hCA IX, except for compound **13**, showcasing a K_I_ of 21.2 nM.

Derivatives **11**–**17** were further assessed for telomerase inhibition activity by a TRAP-based assay at a 10 μM concentration using Colo-205 cell lysates and were compared to BIBR1532 as a positive control. However, only hybrids **12** and **17** induced over 70% inhibition of the telomerase activity in these cell-free system experiments [25]. Consequently, **12** and **17** were further tested on human colon cancer cells, namely, Colo-205, HCT-116, HT-29, and SW-620, to observe their telomerase inhibition (Table 3). Furthermore, the metabolic activities of Colo-205 cells treated with **12** and **17** were assessed by means of the MTT assay after 72 h of incubation. As a result, **12** and **17** showed relatively moderate cytotoxicities, with 15.3% and 35.4% cell viability at a 100 μM concentration, respectively [25].

One year later, in 2021, Berrino et al. proposed the first set of CAI hybrids bearing CO-releasing molecules (CORMs) to counteract inflammatory states, such as RA [26]. The authors reported several coumarin derivatives based on known CAIs. Reported compounds **18**–**20** have been evaluated in vitro both for their selective hCA inhibition and their CO-releasing properties, studying them in the Soret region of the Mb-CO absorption spectra [26]. Derivatives **18**–**20** showed a valuable inhibitory profile against various hCAs, making them selective against hCA XII (Table 4). Particularly, compounds **18** and **20** also exhibited a remarkable inhibition of hCA IX, making them suitable candidates for further studies [26].

In 2023, the same research group proposed an evolution of the previous work [26], pursuing a deeper investigation on CAI−CORM hybrids based on the coumarin scaffold, aiming to explore the influence of various linkers [27]. In this study, the authors investigated 4-, 6-, and 7-substituted coumarins with different alkyne chains and assessed all of them for their ability to release CO and inhibit hCAs I, II, IX, and XII. The results for a selection of the most promising candidates, **21**–**25**, are reported in Table 5. Furthermore, in vivo studies, estimated by means of paw-pressure and incapacitance tests, demonstrated the pain-relieving properties of hybrid compounds **21**–**25** in a rat model of RA, with high potency and long-lasting effects in comparison to those of the reference drug ibuprofen [27].

In 2024, Nocentini et al. proposed a series of multitargeting compounds with the aim of inhibiting cancer-associated CAs and stabilizing the G-quadruplex (GQ), which are noncanonical nucleic acid secondary structures recognised as playing major roles in carcinogenesis [28,29]. In order to achieve selectivity on hCAs IX and XII over I and II, the authors designed a series of coumarins linked with berberine, a known stabilizer of GQ. To synthesize this series, different alkyl chains were appended to the berberine scaffold, and further, Cu(I)-catalyzed Huisgen azide-alkyne cycloaddition was performed in order to provide several coumarin-based hybrids, **26**–**35** (Table 6) [28]. The latter were assayed for their hCA inhibition (Table 6), showing a remarkable selectivity for the cancer-associated CAs IX and XII over the cytosolic I and II. Moreover, these hybrids were screened for their ability to bind to and stabilize DNA GQ-forming sequences, compared to berberine, by circular dichroism (CD) melting experiments, employing three sequences, namely, *Tel*_23_, *c-Kyt1*, and *c-Myc* (Table 6) [28]. The results showed that hybrids were generally more effective than berberine in stabilizing the parallel GQ structures adopted by *c-Kit1* and *c-Myc* over the hybrid *Tel*_23_. Particularly, *c-myc* GQ was strongly stabilized by most coumarin derivatives [28].

In 2024, Braconi et al. proposed a new series of piperazine derivatives bearing a coumarin scaffold with the aim of obtaining dual inhibitors of P-glycoprotein (P-gp), an ABC transporter [30], and hCA XII to synergistically overcome P-gp-mediated multidrug resistance (MDR) in cancer cells [31]. The authors synthesized more than thirty hybrids and assessed all of them for their hCA inhibition using a kinetic assay and for their P-gp activity by evaluating the cytotoxicity enhancement of the co-administered doxorubicin in K562/DOX cells [31]. Inhibition data regarding the compounds **36**–**39** is reported in Table 7. Hybrids **36**–**39** showed a remarkable selectivity for hCAs IX and XII over isoforms I and II. On the other side, **36**–**39** resulted in strong P-gp inhibitors, as demonstrated by RF values higher than 10 (Table 7).

### 2.2. Other Heterocyclic Pharmacophores as Effective Carbonic Anhydrase Inhibitory Chemotypes

In the same publication [26], Berrino et al. proposed two acesulfame-based CAI inhibitors bearing CO-releasing molecules (CORMs) to counteract inflammatory states, such as RA [20]. Reported compounds **40** and **41** have been evaluated in vitro both for their selective hCA inhibition and their CO-releasing properties, studied in the Soret region of the Mb-CO absorption spectra [26]. Derivatives **40** and **41** showed an interesting inhibitory profile against various hCAs, making them selective against hCA XII (Table 8). Particularly, compound **40** also exhibited a remarkable inhibition of hCA IX, making it a suitable candidate for further studies [26]. Successively, the authors have determined their effectiveness on a murine cell line in terms of metabolic activity and proliferation up to 48 h of treatment. Next, these hybrids were tested on LPS-stimulated cells, mimicking inflammatory conditions in vitro. The authors, therefore, observed a counteraction of the inflammatory stimulus at a biological level, mainly with cell metabolic activity restored after 48 h in the presence of compounds **40** and **41**, but also a decreased release of TNF-α [26].

One year later, in 2022, Tan et al. designed two tetrazole-based hybrids, **42** and **43**, and evaluated those compounds against hCAs I and II and xanthine oxidase (XO). The latter is a highly versatile flavoprotein enzyme involved in uric acid formation. Derivatives **42** and **43** showed an interesting inhibitory profile (Table 9), exhibiting micromolar activity against both targets [32].

In 2023, Angeli et al. designed and synthesized a large series of pyridylsulfonamides as multitargeting inhibitors of CAs and agonists of the transient receptor potential vanilloid 1 (TRPV1) receptor [33]. The latter is important as a potential analgesic target since it is involved in the transmission of nociceptive stimuli by triggering an important cellular influx of Ca^2+^ ions [34]. Based on the SB-705498 structure, the authors synthesized more than thirty analogs bearing a sulfonamide group into the pyridine heterocycle. Their inhibitory activity was detected against several hCAs, I, II, IV, VII, IX, and XII, and also their modulation of the TRPV1 receptor activity. Despite the strong inhibition of diverse hCAs, only two compounds, **44** and **45**, resulted in drugs capable of acting as TPRV1 agonists (Table 10) [33]. Furthermore, based on EC_50_ values, derivative **45** was selected as the best compound to be subjected to an in vivo mouse model of neuropathic pain induced by repeated oxaliplatin treatment. Observing a lack of latency, **45** peaked at 30 min post-administration and was effective up to 45 min [33].

## 3. Chimeric Carbonic Anhydrase Inhibitors

Chimeric heterocycle hybrids are a unique class of molecular entities that combine two or more distinct heterocyclic moieties within a single framework. These hybrids are designed to merge the structural and functional attributes of different heterocycles, resulting in novel compounds with enhanced or synergistic biological, chemical, or physical properties.

**Figure 1:**
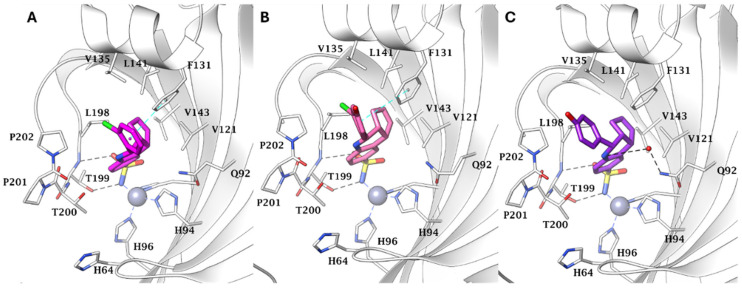
Compounds **46**–**48** in the active site of hCA II. (**A**) Active site region of hCA II/**46** (PDB: 6SX9); (**B**) hCA II/**47** (PDB: 6SYB); and (**C**) hCA II/**48** (PDB: 6SYS).

Regarding this subject, Tinivella et al. proposed a chimeric hCA inhibitor class with the aim of targeting breast cancer by inhibiting both hCAs IX and XII and estrogen receptors (ERs) [35]. In this work, the authors devised a combined ligand-based and structure-based multitarget repurposing strategy and applied it to a series of hexahydrocyclopenta[c]quinoline compounds. In this context, the two components incorporated into the chimeric scaffold were Erteberel (DB07933) [36] and a benzanilide derivative (DB07476) [37], each contributing distinct mechanistic features that collectively improve the therapeutic potential. Among them, Erteberel is an estrogen receptor β-agonist that has been used in trials studying the treatment of Benign Prostatic Hyperplasia, while the benzanilide derivative is a known CA IX inhibitor. The authors synthesized three derivatives, **46**–**48**, and evaluated their activity against hCAs (Table 11), showing that only compound **47** exhibited a notable selectivity for the cancer-associated CAs IX and XII over cytosolic I and II [35]. To compare the effects on ER activity, the authors performed a 3xERE-driven luciferase transactivation assay in agonist mode using HEK-293T cells. In this assay, none of the compounds activated ERα, but **47** and **48** partially activated ERβ compared to the natural estrogen 17β-estradiol. Furthermore, the anticancer activity was evaluated in a cell culture model; however, none of the hybrids exhibited notable antiproliferative activity. Finally, to fully understand the binding mode of derivatives **46**–**48** with hCAs, they were cocrystallized in the active site of hCA II (Figure 1).

## 4. Heterocycles Used as Linkers for the Design of Carbonic Anhydrase Inhibitors

Using heterocyclic rings as linkers between two pharmacophoric units is a simple and highly versatile synthetic strategy in medicinal chemistry. The availability of robust synthetic protocols, coupled with the rapid and flexible incorporation of nitrogen- or oxygen-containing heterocycles as linkers, enables the efficient generation of pharmacophore hybrids. These heterocycles can contribute to conformational rigidity and overall molecular polarity and direct interactions with biological targets, thereby enhancing binding affinity and selectivity. Furthermore, the modular nature of heterocyclic scaffolds enables variation in both substituents and ring systems, thus facilitating the modification of physicochemical and target interaction properties with minimal synthetic effort. Overall, heterocycles used as linkers offer synthetic accessibility and structural diversity while actively participating in binding mechanisms, making them an ideal platform for dual-pharmacophore drug design [38,39,40].

In 2020, Elzahhar et al. reported, for the first time, a novel series of multitarget-directed compounds designed to simultaneously inhibit cyclooxygenase-2, 15-lipoxygenase (15-LOX), and tumor-associated carbonic anhydrases to develop new anticancer agents [41]. The researchers employed a 1,2,3-triazole linker to conjugate distinct pharmacophoric moieties, yielding the hybrid structures depicted in Table 12. Compounds **49**–**60** were subsequently evaluated in vitro for their inhibitory activity against COX-1; COX-2; 15-LOX; and hCA isoforms I, II, IX, and XII (Table 12).

Among them, compounds **57**–**60** exhibited superior COX-2 inhibition compared to the reference drug celecoxib, as well as enhanced COX-1/COX-2 selectivity indices. The entire series demonstrated moderate 15-LOX inhibition, and compounds **57** and **59** showed potent inhibition of the tumor-associated CA isoforms hCA IX and XII. It is noteworthy that the activity of the compounds toward COX-1 and COX-2 is generally comparable within members of the same subseries, suggesting a limited influence of the CAI moiety on COX binding. Conversely, regarding CA inhibition, a sulfonamide group was present, as the CAI moiety usually results in stronger inhibition, an effect that is particularly pronounced against CA I and II. However, exceptions such as derivative 60 highlight that binding to the CA active site is primarily driven by the CAI moiety but is also strongly influenced by the overall molecular architecture. The antiproliferative activity of these compounds was also assessed in vitro against human cancer cell lines, including lung (A549), liver (HepG2), and breast (MCF-7) cells [41]. Notably, **57** exhibited moderate activity against A549 cells (IC_50_ = 28.5 μM), whereas **59** displayed significant inhibitory activity against MCF-7 cells (IC_50_ = 3.2 μM). Importantly, all compounds demonstrated a favorable safety profile, showing limited cytotoxicity against normal human lung fibroblasts (WI-38). Mechanistic investigations revealed that the antitumor activity of **57** was associated with G2/M cell cycle arrest and apoptosis induction, evidenced by upregulation of caspase-9 and Bax expression and concurrent downregulation of Bcl-2 levels. In vivo studies on a xenograft mouse model further confirmed the therapeutic potential of **59**, which significantly reduced tumor volume following treatment. Finally, in silico docking studies supported the experimental findings, highlighting the dual role of the triazole linker: beyond its structural bridging function, it actively participates in target binding by engaging in key interactions within the active sites [41].

The same research group in 2023 further explored the same type of molecular hybrids by applying bioisosteric modifications to celecoxib (compounds **61**–**70**, Table 13) and polmacoxib (compounds **71**–**80**, Table 14) to develop novel anti-inflammatory agents [42,43]. As in their previous study, the compounds were evaluated in vitro for their inhibitory activities against the primary targets, COX-2, 15-LOX, and hCAs IX and XII, as well as against relevant off-targets, COX-1 and the cytosolic isoforms hCAs I and II. Within the first series, compounds **61**, **62**, and **70** emerged as the most potent dual COX-2/15-LOX inhibitors, exhibiting IC_50_ values in the low micromolar range. In the second series, compounds **71**, **75**, and **76** demonstrated the most promising activity profiles. All compounds across both series showed potent inhibition of hCA IX and XII, with IC_50_ values in the nanomolar range [42,43].

Compounds **61** and **62**, which displayed the most favorable in vitro inhibition profiles, were further assessed in vivo to evaluate their anti-inflammatory potential. Both compounds significantly reduced the number of writhing responses in the acetic acid-induced writhing test and demonstrated efficacy in a carrageenan-induced rat paw edema model. Additionally, ELISA assays revealed a marked decrease in serum levels of key pro-inflammatory cytokines, including TNF-α and IL-1β. Similarly, compounds **75** and **76** from the second series also underwent in vivo evaluation and showed promising analgesic and anti-inflammatory effects, confirming their potential as lead candidates for further preclinical development [42,43].

Recently, the research group of Sharma employed heterocyclic linkers, specifically triazole-based structures, for the development of novel anticancer agents targeting tumor-associated carbonic anhydrases (hCAs IX and XII) and cathepsin B [44]. In 2023, a panel of 28 keto-bridged dual-triazole-containing benzenesulfonamides was synthesized and biologically evaluated for inhibitory activity against hCA isoforms and cathepsin B. Selected compounds with the most promising in vitro biological profiles are presented in Table 15. Of these, compound **87** demonstrated the most favorable inhibition profile toward hCAs IX and XII, displaying K_I_ values lower than those of the standard inhibitors **AAZ** and **SLC-0111**. In contrast, compound **89** emerged as the most potent cathepsin B inhibitor within the series but only displayed activity in the millimolar range. In silico molecular docking studies were conducted to explore the binding interactions of the most active derivatives. The results highlighted the key role of the triazole linker in mediating direct target engagement. In the case of cathepsin B, the triazole ring was found to form a specific hydrogen bond with the side chain of His199 within the active site, contributing significantly to binding affinity. In contrast, within the active sites of CAs, the triazole moiety primarily acted as a structural element critical to correctly orient the terminal tail of the molecule, thereby optimizing its interactions with key residues lining the binding cavity [44].

In the following year, Sharma and co-workers continued to refine this dual-inhibitor strategy by incorporating a 1,2,4-triazole linker, resulting in the design and synthesis of 22 novel derivatives [45]. The most active compounds from this new series are shown in Table 16. As observed, the new derivatives retained strong inhibitory activity against hCA IX and XII, with compound **102** exhibiting the most promising profile, although with slightly weaker K_I_ values compared to compound **87** from the previous series. However, the second-generation compounds showed significantly enhanced cathepsin B inhibition, reaching activity in the submicromolar range (10^−7^ M), thus marking a substantial improvement in dual-target efficacy [45].

In 2024, Abbas et al. applied a sugar-tail approach utilizing a 1,2,3-triazole linker to conjugate a benzenesulfonamide moiety with glycosidic fragments, aiming to develop dual inhibitors of tumor-associated carbonic anhydrases (hCAs IX and XII) and vascular endothelial growth factor receptor 2 (VEGFR-2) as novel anticancer agents [46]. The designed and synthesized compounds are presented in Table 17 and were evaluated in vitro for their inhibitory activity against VEGFR-2, hCA IX, and hCA XII, yielding results comparable to those of the standard inhibitors sorafenib and **SLC-0111** [46].

These were further screened for antiproliferative activity against various human cancer cell lines, including lung (A549), liver (HepG2), breast (MCF-7), and colorectal (HCT-116) cancer cells. Both compounds showed notable potency against HepG2 cells (IC_50_ = 10.45 μM for compound **106** and 8.39 μM for compound **107**) and MCF-7 cells (IC_50_ = 20.31 μM for **106** and 21.15 μM for **107**), with activity values comparable to those of doxorubicin (IC_50_ = 13.76 μM and 17.44 μM, respectively). Moreover, molecular docking studies were performed to investigate the binding mechanisms of compounds **106** and **107** within the active sites of VEGFR-2, hCA IX, and hCA XII. These analyses highlighted the direct involvement of the triazole linker in specific interactions with key active site residues, in addition to its structural role as a pharmacophore bridge. Furthermore, the difference in the glycidic moiety between compounds **106** and **107** reduces steric hindrance in the latter, potentially explaining the improved profile observed in the in vitro studies [46].

Collectively, these studies underscore the critical importance of linker selection in the design of hybrid compounds incorporating two pharmacophores. Beyond controlling spatial orientation and inter-pharmacophore distance, appropriately designed linkers, particularly those incorporating heteroatoms, can directly contribute to target binding. As emphasized throughout this review, heteroatom-rich linkers such as triazoles may expand the interaction landscape via hydrogen bond and polar contacts, thus enhancing both affinity and selectivity of the resulting molecular hybrids.

## 5. Heterocycles Used as Second Pharmacophores in Multitargeting Carbonic Anhydrase Inhibitors

In the design of dual-target carbonic anhydrase inhibitors (CAIs), various heterocyclic scaffolds have been effectively used as secondary pharmacophores to enhance isoform selectivity and biological potency.

In 2020, Ceni et al. designed and synthesized a novel series of dual-acting compounds as adenosine A_2A_ receptor (hA_2A_ AR) antagonists and hCA IX and XII inhibitors to develop new anticancer agents [47]. The core scaffold used in this study was based on an 8-amino-6-aryl-2-phenyl-1,2,4-triazolo [4,3-a]pyrazin-3-one structure, originally derived from a known hA_2A_ AR antagonist, which was conjugated with various hCA-inhibiting moieties through different types of linkers. Representative compounds from the series are shown in Table 18. Many of the synthesized derivatives demonstrated nanomolar affinity for hA_2A_ AR, with excellent selectivity over other adenosine receptor subtypes. These derivatives exhibited superior affinity to the reference ligands 5-(N-ethyl-carboxamido)adenosine (NECA) and 2-chloro-N6-cyclopentyladenosine (CCPA). Regarding CA inhibition, most compounds displayed moderate activity, with K_I_ values in the micromolar range. Among them, compound **114** exhibited the most promising inhibition profile toward hCAs IX and XII. Considering the dual activity profile, compounds **114** and **120** emerged as the most promising candidates for further pharmacological evaluation [47].

Molecular docking studies were conducted to elucidate the binding modes of selected derivatives within both targets. Interestingly, although the CAI moiety does not contribute to interactions with the hA_2A_ receptor, the triazolopyrazinone core was found to be directly involved in binding to the outer region of the hCA active sites. Specifically, the nitrogen atom of the pyrazine ring forms a hydrogen bond with the hydroxyl group of Ser132 in hCA XII [47].

These findings highlight the importance of rational design in dual-target agents, where both pharmacophoric units must be considered active contributors to binding. The optimal spatial and electronic complementarity of both moieties is essential to maximize interactions across the distinct binding environments of the two targets.

In the field of anticancer drug discovery, Zhang and co-workers developed a series of dual inhibitors targeting both the epidermal growth factor receptor (EGFR) and tumor-associated hCA IX by combining quinazoline-based derivatives with benzenesulfonamide moieties [48]. The synthesized compounds were evaluated in vitro for their inhibitory activity against wild-type EGFR (EGFR^WT^), where compound **123** demonstrated superior potency compared to the reference drug gefitinib. Against the EGFR^T790M^ mutant, compound **124** exhibited significantly improved inhibition, achieving activity levels comparable to osimertinib (Table 19). Additionally, the compounds were tested for their inhibitory activity against hCA II and hCA IX, with compound **124** again emerging as the most potent and selective inhibitor of the tumor-associated isoform hCA IX.

The antiproliferative activity of the compounds was assessed in vitro against various human cancer cell lines, including epidermoid carcinoma (A431) and non-small cell lung cancer (A549 and H1975). Notably, compound **124** exhibited comparable potency to osimertinib in H1975 cells (IC_50_ = 1.94 μM vs. 0.98 μM) and even outperformed the reference drug under hypoxic conditions (IC_50_ = 1.05 μM vs. 2.08 μM). Western blot analysis revealed that **124** significantly suppressed the expression of phosphorylated EGFR (p-EGFR) and its downstream signaling proteins p-AKT and p-ERK in H1975 cells. Furthermore, under hypoxic conditions, it also inhibited the expression of hCA IX and its upstream regulator HIF-1α, confirming its dual mechanism of action. Finally, molecular docking studies provided further insight into the binding interactions of compound **124** with hCA IX, EGFR^WT^, and EGFR^T790M^. In EGFR^WT^, the sulfonamide moiety forms a key hydrogen bond with Lys745. In the EGFR^T790M^ mutant, the altered binding pocket architecture allows for two additional hydrogen bonds between the sulfonamide group and residues Arg841 and Asp855. These enhanced interactions may explain the potent inhibitory activity of **124** against the resistant EGFR^T790M^ kinase variant [48].

In 2024, Giovannuzzi et al. designed and synthesized a series of dual inhibitors targeting brain hCAs and Monoamine Oxidase B (MAO-B), with the aim of modulating multiple pathological pathways implicated in Alzheimer’s disease and preventing β-amyloid (Aβ-42)-associated neurotoxicity [49]. The authors combined a chromone or coumarin scaffold, known for its reversible inhibition of MAO-B, with a benzenesulfonamide moiety to target hCAs. Initially, four distinct series of hybrid compounds (Series 1–4, Table 20) were developed and evaluated for their inhibitory activity against hCA isoforms I, II, IV, VA, VB, VII, and XII, as well as hMAO-A and hMAO-B. The results were benchmarked against standard drugs including methazolamide (**MTZ**), acetazolamide (**AAZ**), clorgyline (**CLO**), and selegiline (**SEL**). Among the tested compounds, derivatives from Series 3 demonstrated the most favorable dual-target profile, showing potent inhibition of both hCA isoforms and hMAO-B, with K_I_ values in the low nanomolar range [49].

Encouraged by these findings, the authors expanded the library with a fifth series (Series 5), although no further improvement in inhibitory potency was observed. Nevertheless, the most promising multitarget compounds from the earlier series effectively mitigated Aβ-42-induced toxicity, significantly reducing reactive oxygen species (ROS) levels and restoring mitochondrial function in SH-SY5Y neuroblastoma cells [49].

Gamal and coworkers designed and synthesized a series of benzenesulfonamide–thiazolidinone hybrids as novel multitarget agents for the management of type 2 diabetes mellitus [50]. These agents simultaneously inhibit key enzymes, such as α-glucosidase (targeted by the thiazolidinone moiety) and hCA II (targeted by the benzenesulfonamide moiety). All synthesized derivatives exhibited notable α-glucosidase inhibitory activity, with compounds **131**, **133**, **134**, and **140** showing comparable potency to the reference drug acarbose. Among these, compound **134** emerged as the most potent, displaying higher inhibitory activity than acarbose. Regarding carbonic anhydrase inhibition, compound **133** demonstrated the strongest inhibitory effect against the target hCA II, surpassing the standard inhibitor **AAZ**. In addition, the compounds were tested against the tumor-associated hCA isoforms IX and XII, with compound **144** showing the most promising inhibitory profile in this subset (Table 21).

Given the encouraging in vitro results, compound **133** was selected for in vivo glucose tolerance testing to evaluate its hypoglycemic effect on diabetic mice. Interestingly, compound **133** significantly reduced blood glucose levels compared to acarbose, supporting its potential as an antidiabetic agent [50].

Furthermore, the antiproliferative activity of the most active hCA IX/XII inhibitors, compounds **140**, **141**, and **144**, was assessed in MCF-7 breast cancer cells under both normoxic and hypoxic conditions. Compound **144** exhibited the highest efficacy under normoxic conditions (IC_50_ = 7.95 µM), while compound **140** was more effective under hypoxia (IC_50_ = 14.83 µM). However, both compounds were less potent than the reference drug doxorubicin, which showed IC_50_ values of 3.84 µM and 9.43 µM, respectively [50].

In 2024, Bonardi et al. designed and synthesized a novel series of dual-acting antibiotics in response to the growing challenge of antibiotic resistance [51]. The strategy involved conjugating a benzenesulfonamide-based CAI moiety to well-known β-lactam antibiotics such as ampicillin and amoxicillin. The synthesized hybrids were initially evaluated in vitro for their inhibitory activity against three bacterial CA isoforms, CynT2 (β-CA from *Escherichia coli*), EcoCA (γ-CA from *E. coli*), and NgCA (α-CA from *Neisseria gonorrhoeae*), as well as against the human off-target isoforms hCA I and hCA II. Most compounds demonstrated strong inhibition of the bacterial CAs, consistently outperforming the reference inhibitor **AAZ**. Specifically, compounds **145**, **146**, and **158** were the most potent against CynT2; **148**, **149**, and **151** were the most potent against EcoCA; and **146**, **158**, and **163** were the most potent against NgCA (Table 22).

Since NgCA was identified as the most selectively inhibited bacterial isoform, the authors further investigated the antibacterial activity of the compounds against clinical strains of *N. gonorrhoeae*, including multidrug-resistant strains, both β-lactam-sensitive and resistant to ceftriaxone and/or azithromycin. Notably, compounds **145** and **161** showed 4- to 8-fold enhanced activity compared to ampicillin and amoxicillin against the *N. gonorrhoeae* FA1090 strain, with MIC values of 0.03 µM for both **145** and **161**, compared to 0.125 µM and 0.250 µM for ampicillin and amoxicillin, respectively. Finally, the binding potential of the β-lactam portion for penicillin-binding proteins (PBPs) was assessed via in silico covalent docking studies, which suggested that, in several compounds, the benzenesulfonamide moiety also contributed directly to interactions with active site residues of PBPs [51].

In 2025, Lopez’s research group developed a novel class of sulfonamide–thiosemicarbazone hybrids designed to combine metal chelation and hCA inhibition as a dual approach for anticancer therapy [52]. The compounds were evaluated for their inhibitory activity against hCAs I, II, IX, and XII, showing good potency and notable selectivity toward the tumor-associated isoforms hCAs IX and XII (Table 23).

Within this series of compounds, **166** emerged as the most potent dual inhibitor of hCAs IX and XII, underscoring that the positioning of the sulfonamide group (meta rather than para) exerts a greater influence than the linker length. Given its promising profile, its metal-chelating properties were investigated further against a range of metal ions, including Na^+^, K^+^, Fe^2+^, Fe^3+^, Zn^2+^, and Cu^2+^. Compound **166** showed no affinity for monovalent cations but demonstrated a strong ability to chelate Cu^2+^, suggesting potential for disrupting metal-dependent tumor processes. The antiproliferative activity of derivatives was then assessed in vitro against a panel of cancer cell lines, including lung (A549, SW1573), breast (HBL-100, T-47D), cervical (HeLa), and colon (WiDr) cells. Notably, compound **166** exhibited the most potent antiproliferative effects, with GI_50_ values ranging from 4.5 to 10 µM, reinforcing its potential as a multifunctional anticancer agent [52].

In the same year, Elkotamy and co-workers designed and synthesized a series of pyrazolo[1,5-a]pyrimidine derivatives bearing zinc-binding groups to achieve dual inhibition of tumor-associated carbonic anhydrase isoforms IX and XII, as well as cyclin-dependent kinase 6 (CDK6), targeting critical pathways in non-small-cell lung cancer (NSCLC) [53].

The synthesized derivatives, reported in Table 24, were evaluated in vitro for their inhibitory activity against hCAs I, II, IX, and XII. The majority of compounds displayed potent inhibition and marked selectivity toward hCAs IX and XII, with compounds **170**, **178**, **180**, and **183** emerging as the most selective toward the tumor-associated isoforms over the cytosolic hCA I and hCA II. The results indicate that CA inhibition is primarily influenced by the type of CA inhibitor (R_1_), with sulfonamide derivatives showing superior activity compared to their acidic counterparts. Substituents at positions R_2_ and R_3_ exert variable effects across the four isoforms. In general, the presence of fluorine at R_2_ enhances inhibitory activity against all four tested isoforms, whereas the methoxy group (OCH_3_) appears to be optimal for improving the selectivity of the target isoforms CA IX and CA XII. The influence of R_3_ is more limited; however, the hydroxyl group enhances inhibition of CA I while reducing activity against CA II, IX, and XII compared to the methyl substituent.

The compounds were subsequently assessed for their cytotoxic effects on NSCLC cell lines. Notably, compounds **170**, **171**, **176**, and **181** exhibited superior potency compared to the reference CDK inhibitor roscovitine. Specifically, the following IC_50_ values were recorded:

- In A549 cells: 1.90 μM (**170**), 5.98 μM (**171**), 4.91 μM (**176**), and 2.39 μM (**181**) versus 15.91 μM for roscovitine.
- In NCI-H1734 cells: 5.45 μM (**170**), 0.75 μM (**171**), 2.02 μM (**176**), and 7.92 μM (**181**) versus 9.10 μM for roscovitine.

The same four compounds were further evaluated for CDK4/6 inhibition, revealing preferential activity toward CDK6. Moreover, mechanistic studies demonstrated that the compounds induced cell cycle arrest at the G1 phase and promoted apoptosis. Compound **171** increased the G1 phase population to 91.02% in NCI-H1734, while **181** achieved 83.66% in A549 cells. In cytotoxicity assays against normal WI-38 fibroblasts, compound **171** showed an IC_50_ of 30.80 μM, and **181** exhibited 58.87 μM, both showing acceptable safety margins when compared to staurosporine (IC_50_ = 19.59 μM) [53].

## 6. Conclusions

The integration of heterocycles into multitarget carbonic anhydrase inhibitors (CAIs) represents a recent but dynamic and rapidly evolving strategy in drug discovery. Heterocyclic motifs, owing to their inherent versatility, can act as core inhibitory chemotypes, structural linkers, or complementary pharmacophores, thus offering multiple entry points for rational molecular design. By enabling the simultaneous modulation of different biological targets, including multiple hCA isoforms, these compounds address the inherent complexity of multifactorial diseases such as cancer, glaucoma, inflammatory disorders, and neurodegenerative conditions. The multitarget approach is especially helpful for diseases caused by connected biochemical pathways because it can lower the need for many drugs, improve treatment results, and reduce side effects by using one treatment that works on multiple parts.

In this context, the use of (sulfo)coumarin cores as CAIs represents the most used strategy to selectively target the cancer-associated hCAs IX and XII, avoiding inhibition of the cytosolic and off-target hCAs I and II. Moreover, many of these (sulfo)coumarin-based hybrids were assessed for their antiproliferative effects, showing favorable results, as demonstrated by derivatives **11**–**17**, targeting both hCAs IX and XII and telomerase [25]. Furthermore, several publications have also investigated coumarin-related anti-inflammatory activity [26,27], as discussed for compounds **20**–**27**. Among these, **23**–**27** exhibited consistent pain-relieving properties in an in vivo model [27]. However, the heterocycle’s role in hCA inhibition has also been widely explored, particularly as a linker, because of its ability to establish various interactions with surrounding amino acid residues (e.g., hydrogen bonds and π-π interactions) and for its facile and rapid synthesis. A notable example is Cu(I)-catalysed Huisgen azide–alkyne cycloaddition, which forms the 1,2,3-triazole core. This approach has been extensively adopted to connect two active pharmacophores in a multitargeting strategy [24,28,41,44,46].

In general, hybrid and chimeric heterocyclic designs have proven especially valuable, allowing for major physicochemical properties, optimizing binding affinities, and enhancing isoform selectivity. Advances in computational chemistry, high-throughput screening, and structure-guided drug design are now promoting the identification of promising heterocyclic scaffolds, as well as the prediction of off-target interactions and potential toxicity. Accordingly, Tinivella et al. used in silico studies of the ligand-based and structure-based multitarget repurposing strategy to discover new chimeric inhibitors, **46**–**48** [35]. They were able to effectively modulate both hCAs and estrogen receptors, confirming the valuable role of computational chemistry in the design of consistent inhibitors. Future progress will rely on the integration of medicinal chemistry with structural biology and fragment-based screening to better understand target networks and refine ligand design.

Ultimately, the rational exploitation of heterocycles in multitarget paradigms holds the potential to generate next-generation therapeutics with enhanced efficacy and improved patient compliance, thereby paving the way for innovative treatments of complex diseases.

## Figures and Tables

**Table 1:** hCA I, II, IV, IX, and XII inhibition data with compounds **1**–**10** using acetazolamide (**AAZ**) as a reference drug.

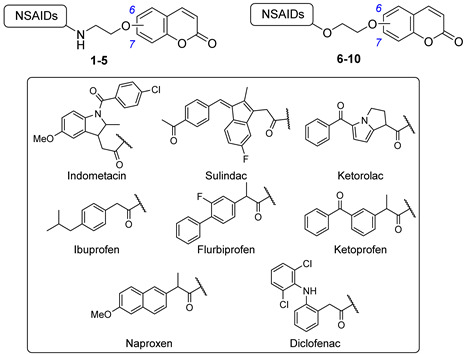							
Cmp	Linker Position (*6-*/*7-*)	NSAID	K_I_ (nM)				
			hCAs				
			I	II	IV	IX	XII
**1**	*6*	Indometacin	>100,000	>100,000	2.6	31.3	59.1
**2**	*7*	Sulindac	>100,000	>100,000	9.0	27.9	7.7
**3**	*7*	Ibuprofen	>100,000	>100,000	9.1	>100,000	39.0
**4**	*7*	Flurbiprofen	>100,000	>100,000	8.8	>100,000	>100,000
**5**	*7*	Ketorolac	>100,000	>100,000	9.4	>100,000	>100,000
**6**	*6*	Ketoprofen	>10,000	>10,000	2.3	36.9	83.1
**7**	*6*	Indometacin	>10,000	>10,000	3.7	27.7	51.5
**8**	*7*	Diclofenac	>10,000	>10,000	4.3	4.5	88.1
**9**	*7*	Ketoprofen	>10,000	>10,000	6.7	35.4	55.4
**10**	*7*	Naproxen	>10,000	>10,000	6.3	13.3	73.7
**AAZ**	-	-	250.0	12.0	74.2	25.0	5.7

**Table 2:** Inhibition data for hCAs I, II, IX, and XII with compounds **11**–**17** using the standard drug **AAZ** in a stopped-flow CO_2_ hydrase assay.

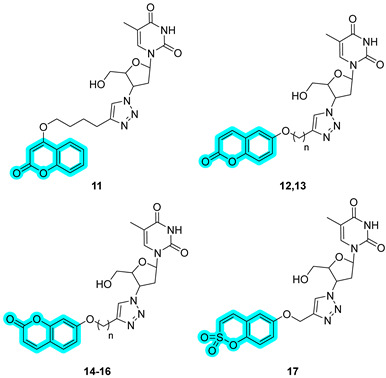					
Cmp	n	K_I_ (nM)			
		hCAs			
		I	II	IX	XII
**11**	-	>10,000	>10,000	>10,000	8.7
**12**	1	>10,000	>10,000	6557	3.6
**13**	3	>10,000	>10,000	21.2	9.4
**14**	1	>10,000	>10,000	4885.7	3.5
**15**	3	>10,000	>10,000	2948	40.0
**16**	4	>10,000	>10,000	>10,000	8.9
**17**	-	>10,000	>10,000	5852	2.8
**AAZ**	-	250.0	12.0	25.0	5.7

**Table 3:** IC_50_ and IC_90_ (μM) values for the telomerase inhibition of compounds **13** and **17** in human colon cancer cell lines Colo-205, HCT-116, HT-29, and SW-620.

Cmp	IC_50_ [IC_90_] (μM)			
	Colo-205	HCT-116	HT-29	SW-620
**12**	14.8 [67.5]	5.7 [32.1]	28.6 [78.5]	32.4 [96.1]
**17**	19.0 [71.5]	48.4 [174.1]	65.0 [205.8]	75.4 [211.6]

**Table 4:** Inhibition data for hCAs I, II, IX, and XII against compounds **18**–**20**.

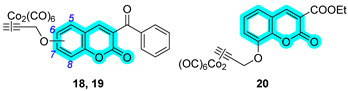					
Cmp	Linker	K_I_ (nM)			
		hCAs			
		I	II	IX	XII
**18**	6	>10,000	>10,000	8112	332.3
**19**	8	>10,000	>10,000	>10,000	4365
**20**	-	>10,000	>10,000	802.6	540.1
**AAZ**		250	12.0	25.0	5.7

**Table 5:** Inhibition data for hCAs I, II, IX, and XII against compounds **21**–**25**.

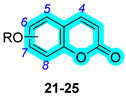						
Cmp	Linker	R	K_I_ (nM)			
			hCAs			
			I	II	IX	XII
**21**	4	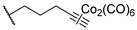	>10,000	>10,000	8.9	8.5
**22**	6	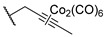	>10,000	>10,000	8.5	5.6
**23**	6	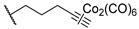	>10,000	>10,000	14.8	6.2
**24**	7	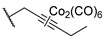	>10,000	>10,000	8.4	8.0
**25**	7	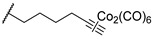	>10,000	>10,000	31.0	2.7
**AAZ**	-	-	250.0	12.0	25.0	5.7

**Table 6:** Inhibition data for human CAs I, II, IX, and XII with compounds **26**–**35** and changes in the GQ-stabilizing effect (ΔΔT_1/2_) of the indicated compounds.

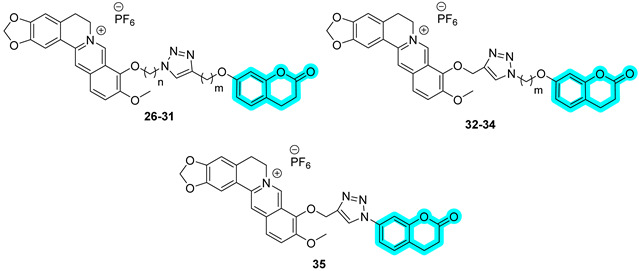									
Cmp	n	m	K_I_ (nM)				ΔΔT_1/2_ (°C) ^a^		
			hCAs						
			I	II	IX	XII	*Tel*_23_	*c-Kyt1*	*c-Myc*
**26**	3	1	>100,000	>100,000	66.0	30.1	1.0	7.0	6.0
**27**	3	2	>100,000	>100,000	34.5	18.9	−1.5	5.5	6.5
**28**	3	3	>100,000	>100,000	58.9	28.4	3.0	4.0	3.0
**29**	4	1	>100,000	>100,000	17.3	8.4	−0.5	4.5	1.0
**30**	4	2	>100,000	>100,000	30.1	16.7	−2.0	4.5	3.0
**31**	4	3	>100,000	>100,000	48.6	20.1	−1.0	7.0	>13.0
**32**	-	3	>100,000	>100,000	35.1	10.2	1.0	4.0	4.0
**33**	-	4	>100,000	>100,000	25.5	4.2	−2.5	3.5	2.0
**34**	-	5	>100,000	>100,000	9.6	14.5	−2.0	4.5	>13.0
**35**	-	-	>100,000	>100,000	51.5	5.4	−2.5	3.5	>13.0
**AAZ**	-	-	250	12.0	25.0	5.7	-	-	-

^a^ ΔΔT_1/2_ values are the difference between ΔT_1/2_ induced by the hybrid compounds and those induced by berberine.

**Table 7:** Inhibitory effects on hCA I, II, IX, and XII isoforms and doxorubicin cytotoxicity enhancement effect on K562/DOX cells of compounds **36**–**39**.

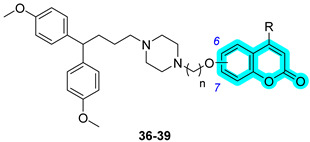									
Cmp	R	Linker (*6-*/*7-*)	n	K_I_ (nM)				RF ^a^	
				hCAs					
				I	II	IX	XII	1 μM	3 μM
**36**	H	7	4	>100,000	>100,000	145	92.1	8.1	90.5
**37**	Me	7	4	>100,000	>100,000	124	82.4	7.7	25.5
**38**	H	6	4	>100,000	>100,000	74.6	46.8	9.9	31.7
**39**	Me	6	3	>100,000	>100,000	523	335	10.4	14.3
**AAZ**	-	-	-	250	12.0	25.0	5.7	-	-
**Verapamil**	-	-	-	-	-	-	-	1.2	3.0

^a^ Inhibition of P-gp transport activity in K562/DOX cells expressed as RF, that is, the ratio between the IC_50_ of doxorubicin alone and in the presence of modulators (RF = IC_50_ of doxorubicin–modulator/IC_50_ of doxorubicin + modulator).

**Table 8:** Inhibition data for hCAs I, II, IX, and XII against compounds **40** and **41**.

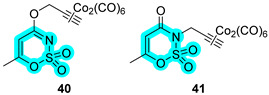					
Cmp	Linker	K_I_ (nM)			
		hCAs			
		I	II	IX	XII
**40**	-	>10,000	>10,000	56.3	788.4
**41**	-	>10,000	>10,000	>10,000	3462
**AAZ**		250	12.0	25.0	5.7

**Table 9:** Enzyme inhibition results for compounds **42** and **43** against hCAs I and II and xanthine oxidase.

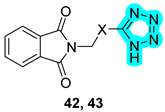				
Cmp	X	IC_50_ (μM)		
		hCA I	hCA II	XO
**42**	-CH_2_S-	6.13	5.63	13.64
**43**	-CH_2_-	4.16	3.87	5.31

**Table 10:** In vitro data for compounds **44** and **45** against hCAs I, II, IV, VII, IX, and XII, and for their ability to modulate the TPRV1 receptor.

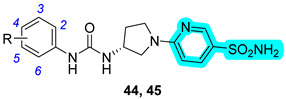								
Cmp	R	K_I_ (nM)						EC_50_ (μM)
		hCAs						TPRV1
		I	II	IV	VII	IX	XII	
**44**	3-CF_3_,4-Cl	788.1	453.4	4019	78.6	737.0	67.6	74.5
**45**	2-Cl	82.5	70.3	3002	73.5	316.2	83.6	11.9
**AAZ**	-	250.0	12.0	74.2	2.5	25.0	5.7	-

**Table 11:** Inhibitory effects of synthesized hexahydrocyclopenta[c]quinoline compounds on different hCAs.

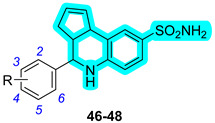					
Cmp	R	K_I_ (nM)			
		hCAs			
		I	II	IX	XII
**46**	2-Cl	73.6	2.2	2089	255.8
**47**	2-Cl,4-OH	706.2	539.1	56.3	78.8
**48**	4-OH	6.7	94.6	803.5	346.2
**AAZ**	-	250	12.0	25.0	5.7

**Table 12:** Enzyme inhibition results for compounds **49**–**60** against hCAs I, II, IX, and XII; cyclooxygenases 1 and 2; and 15-lipoxygenase. Acetazolamide (**AAZ**), celecoxib (CEL), indomethacin (IND), diclofenac (DIC), and quercetin (QER) are reported as reference compounds.

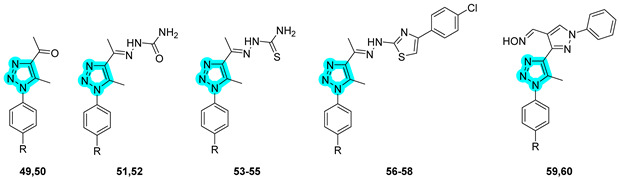									
Cmp	R	K_I_ (nM)				IC_50_ (µM)			SI COX-1/COX-2
		hCA I	hCA II	hCA IX	hCA XII	COX-1	COX-2	15-LOX	
**49**	SO_2_NH_2_	73.0	7.9	9118	82.6	7.6	0.11	3.65	69.1
**50**	SO_2_NH_2_-thiazole	51,001	6280	>100,000	>100,000	5.8	0.29	4.72	20.0
**51**	SO_2_NH_2_	212.6	16.4	4218	19.8	5.9	0.42	4.74	14.0
**52**	SO_2_NH_2_-thiazole	46,328	6864	>100,000	>100,000	7.9	0.34	6.52	23.2
**53**	COOH	>100,000	416.1	>100,000	>100,000	11.6	0.05	1.65	232.0
**54**	SO_2_NH_2_	83.5	31.9	7270	15.3	13.0	0.05	1.51	260.0
**55**	SO_2_NH_2_-thiazole	>100,000	62,533	>100,000	>100,000	10.3	0.05	1.69	206.0
**56**	COOH	550.8	565.7	>100,000	>100,000	10.9	0.07	1.76	155.7
**57**	SO_2_NH_2_	252.0	14.1	7893	13.4	12.4	0.04	1.34	310.0
**58**	SO_2_NH_2_-thiazole	>100,000	>100,000	>100,000	>100,000	14.2	0.04	1.19	355.0
**59**	COOH	4626	239.2	2254	154.4	12.4	0.04	1.29	310.0
**60**	SO_2_NH_2_	18,340	7710	>100,000	>100,000	13.9	0.04	1.91	347.5
**CEL**	-	50,000	21	-	-	14.7	0.05	-	294.0
**IND**	-	-	-	-	-	0.04	0.49	-	0.08
**DIC**	-	-	-	-	-	3.9	0.8	-	4.9
**QUE**	-	-	-	-	-	-	-	3.34	-
**AAZ**	-	250	12.1	25.8	5.7	-	-	-	-

**Table 13:** Enzyme inhibition results for compounds **61**–**70** against hCAs I, II, IX, and XII; cyclooxygenases 1 and 2; and 15-lipoxygenase. Rofecoxib (ROF) and zileuton (ZIL) are reported as reference compounds.

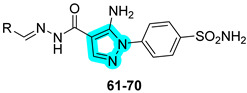									
Cmp	R	K_I_ (nM)				IC_50_ (µM)			*SI* COX-1/COX-2
		hCA I	hCA II	hCA IX	hCA XII	COX-1	COX-2	15-LOX	
**61**	C_6_H_5_	183.4	81.4	38.4	21.6	10.4	0.049	2.4	212.2
**62**	4-OH-C_6_H_4_	133.5	26.9	48.9	5.8	12.5	0.06	1.9	208.3
**63**	4-OCH_3_-C_6_H_4_	279.2	43.5	28.1	21.9	6.0	0.150	4.8	40
**64**	4-N(CH_3_)_2_-C_6_H_4_	81.0	63.8	30.9	11.4	7.0	0.08	4.8	87.5
**65**	2-Cl-C_6_H_4_	368.4	56.7	16.5	44.8	10.5	0.130	3.8	80.8
**66**	4-Cl-C_6_H_4_	105.8	22.7	13.0	24.7	8.0	0.09	4.6	88.9
**67**	2-NO_2_-C_6_H_4_	265.3	33.5	24.1	36.1	4.8	0.140	3.4	34.3
**68**	3-NO_2_-C_6_H_4_	409.7	6.2	65.2	62.0	5.0	0.100	3.5	50
**69**	4-NO_2_-C_6_H_4_	156.4	37.9	82.1	30.4	5.0	0.156	5.0	32.1
**70**	2-thienyl	524.7	68.1	45.8	31.6	9.5	0.06	2.5	158.3
**ROF**		-	-	-	-	14.5	0.027	-	542.5
**ZIL**		-	-	-	-	-	-	3.5	-

**Table 14:** Enzyme inhibition results for compounds **71**–**80** against hCAs I, II, IX, and XII; cyclooxygenase 1 and 2; and 15-lipoxygenase.

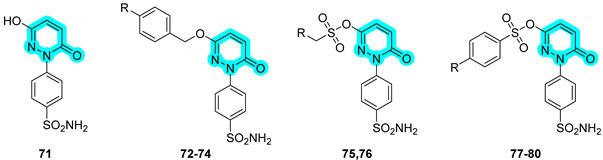									
Cmp	R	K_I_ (nM)				IC_50_ (µM)			*SI* COX-1/COX-2
		hCA I	hCA II	hCA IX	hCA XII	COX-1	COX-2	15-LOX	
**71**		23.5	55.8	45.1	5.3	11.5	0.07	2	167.7
**72**	H	98.3	5.3	14.8	32.2	5	0.11	7	46
**73**	NO_2_	116.3	37.1	12.3	26.1	9.5	0.14	5	67.9
**74**	CN	221.5	106.4	4.9	18.4	6.5	0.07	7	92.9
**75**	H	48.3	42.2	52.3	13.3	10.4	0.05	3	208
**76**	CH_3_	52.6	79.1	58.1	17.2	12.6	0.06	2.5	210
**77**	H	185.9	34.2	19.4	49.7	8.4	0.08	4	106
**78**	CH_3_	123.5	19.2	22.8	42.6	8.4	0.08	3.5	105.9
**79**	OCH_3_	362.8	88.2	30.1	35.9	6.5	0.09	5	73.1
**80**	F	165.8	31.6	6.4	8.7	8.5	0.11	6.5	81

**Table 15:** In vitro K_I_ values of compounds **81**–**92** against hCAs I, II, IX, and XII and % inhibition for cathepsin B. Acetazolamide (**AAZ**), **SLC-0111**, and curcumin are reported as reference compounds.

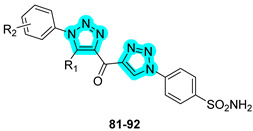							
Cmp	R_1_	R_2_	K_I_ (nM)				% Inhibition Cathepsin B ^a^
			hCA I	hCA II	hCA IX	hCA XII	
**81**	H	H	492.9	4.1	6.1	4.4	53.46
**82**	H	4-CH_3_	756.4	57.5	4.0	2.7	55.49
**83**	H	4-F	650.0	54.6	55.1	33.5	56.28
**84**	H	4-Br	4138	265.3	94.7	64.1	55.23
**85**	H	4-NO_2_	32,771	47.5	70.7	84.6	61.29
**86**	H	4-CN	665.0	8.5	7.7	15.4	63.42
**87**	H	3-OCH_3_	5616	55.1	5.8	0.8	52.48
**88**	H	3-Cl	579.9	22.3	24.8	5.1	55.48
**89**	H	3-NO_2_	817.9	34.6	37.2	71.0	74.28
**90**	H	2-OCH_3_	1508	79.0	52.8	66.9	53.24
**91**	H	2-NO_2_	7953	9.1	281.5	100.9	67.43
**92**	CH_3_	2-NO_2_	888.7	2.9	329.6	90.5	58.08
**AAZ**	-	-	250	12.0	25.0	5.7	76.59
**SLC-0111**	-	-	5080	96	45.1	4.5	-
**Curcumin**	-	-	-	-	-	-	100

^a^ The compounds were tested at 1 × 10^−3^ M concentration; curcumin was tested at 1 × 10^−5^ M concentration.

**Table 16:** In vitro K_I_ values of compounds **93**–**105** and acetazolamide (**AAZ**) against hCA I, II, IX, and XII and % inhibition of cathepsin B.

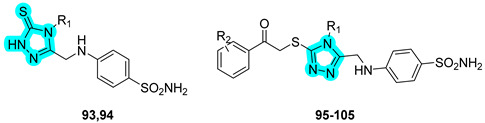							
Cmp	R_1_	R_2_	K_I_ (nM)				% Inhibition Cathepsin B ^a^
			hCA I	hCA II	hCA IX	hCA XII	
**93**	H		353.7	3.4	84.1	11.9	28.32
**94**	C_6_H_5_		4001	22.9	83.3	26.4	40.38
**95**	H	4-OCH_3_	786.8	55.5	78.1	45.3	37.42
**96**	H	4-Br	6627	81.2	245.5	41.2	39.19
**97**	H	4-NO_2_	2829	27.2	160.6	66.5	49.85
**98**	H	3-Cl	968.8	71.0	148.9	132.2	38.17
**99**	H	3-Br	3011	69.0	96.4	37.8	42.42
**100**	C_6_H_5_	4-CH_3_	6622	157.2	99.0	87.2	41.34
**101**	C_6_H_5_	4-OCH_3_	1611	246.7	68.3	21.7	45.27
**102**	C_6_H_5_	4-Cl	733.4	83.3	35.3	16.1	36.14
**103**	C_6_H_5_	4-NO_2_	2994	71.1	45.6	51.1	57.42
**104**	C_6_H_5_	3-Cl	3142	81.2	78.8	54.2	40.24
**105**	C_6_H_5_	3-NO_2_	483.1	238.9	69.1	72.6	51.59
**AAZ**			250	12.0	25.0	5.7	49.17

^a^ The compounds were tested at 1 × 10^−7^ M concentration.

**Table 17:** In vitro K_I_ values of compounds **106** and **107** against hCA IX and XII, and IC_50_ values for VEGFR-2. Sorafenib and **SLC-0111** are reported as reference drugs.

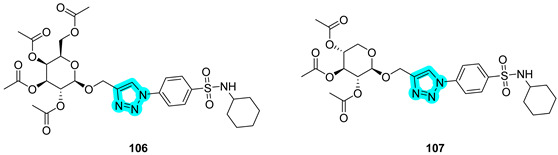			
Cmp	K_I_ (nM)		IC_50_ (µM) VEGFR-2
	hCA IX	hCA XII	
**106**	66	7.6	1.33
**107**	40	3.2	0.38
**Sorafenib**	-	-	0.43
**SLC-0111**	53	4.8	-

**Table 18:** In vitro K_I_ values of compounds **108**–**122** against hCA I, II, IX, and XII and human adenosine receptors 1, 2a, and 3. Acetazolamide (**AAZ**), 5-(N-ethyl-carboxamido)adenosine (NECA) and 2-chloro-N6-cyclopentyladenosine (CCPA)are reported as reference ligands.

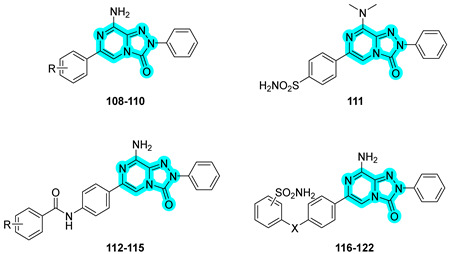									
Cmp	R	X	K_I_ (µM)				K_I_ (nM)		
			hCA I	hCA II	hCA IX	hCA XII	hA_1_	hA_2a_	hA_3_
**108**	2-OH	-	>100	19.0	>100	>100	16.3	2.4	44.5
**109**	3-OH	-	>100	93.0	>100	>100	14	3.5	134
**110**	4-SO_2_NH_2_	-	8.023	0.703	8.920	0.602	205	856.6	14,830
**111**	-	-	2.093	0.464	6.729	0.358	435.3	3886.5	19,495
**112**	4-OCH_3_	-	>100	>100	>100	>100	417.7	0.7	279
**113**	3,4-di OH	-	>100	31.7	44.3	>100	545.7	9	1248
**114**	4-SO_2_NH_2_	-	8.351	0.046	0.466	0.006	4189	6.4	>30,000
**115**	3-SO_2_NH_2_	-	2.288	0.367	0.810	0.303	1211	13.5	>30,000
**116**	4-SO_2_NH_2_	–CH_2_NH–	570.3	896.8	5968	624.9	87.7	27.9	>30,000
**117**	4-SO_2_NH_2_	–COCH_2_NH–	588.7	246.3	9450	25.7	131	26	6760
**118**	4-SO_2_NH_2_	–(CH_2_)_2_NH(CH_2_)_2_CONH–	8713	526.5	108.8	26.3	1031	10.6	5833
**119**	4-SO_2_NH_2_	–CH_2_NH(CH_2_)_2_CONH–	9001	6763	318.7	41.5	839	96.3	>30,000
**120**	4-SO_2_NH_2_	–CONH(CH_2_)_2_CONH–	51.5	8.6	5.0	27.0	1074	108	>30,000
**121**	3-SO_2_NH_2_	–CONH(CH_2_)_2_CONH–	7277	341.0	6925	830.0	418	9.2	>30,000
**122**	4-SO_2_NH_2_	–CONH(CH_2_)_2_O–	933.9	183.4	5951	528.3	420.4	6.8	6645
**AAZ**	-	-	0.25	0.012	0.025	0.005	-	-	-
**NECA**	-	-	-	-	-	-	4.6	16	12.8
**CCPA**	-	-	-	-	-	-	1.2	2050	26

**Table 19:** In vitro IC_50_ values of compounds **123**–**126** against hCA II and IX and epidermal growth factor receptor wild-type (EGFR^WT^) and T790M mutant (EGFR^T790M^). Acetazolamide (**AAZ**), geftinib, and osimertinib are reported as reference ligands.

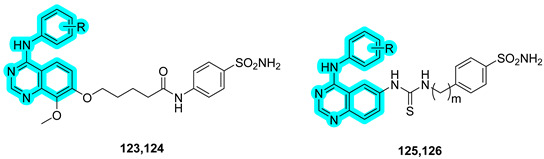							
Cmp	m	R	IC_50_ (nM)				
			hCA II	hCA IX	SI hCA II/hCA IX	EGFR^WT^	EGFR^T790M^
**123**	-	3-CF_3_; 4-Cl	355.8	224.3	1.6	13.7	37.5
**124**	-	3-CF_3_	278.2	115.0	2.4	27.0	9.2
**125**	0	3-CCH	526.2	577.5	0.9	51.2	135.0
**126**	2	3-CCH	241.5	312.8	0.8	42.6	93.4
**Geftinib**	-	-	-	-	-	17.1	378.4
**Osimertinib**	-	-	-	-	-	58.2	8.1
**AAZ**	-	-	45.1	87.2	0.5	-	-

**Table 20:** In vitro K_I_ values of compounds developed by Giovannuzzi et al. against hCAs I, II, IV, VA, VB, VII, and XII and IC_50_ values for Monoamine Oxidases A and B. Methazolamide (**MTZ**), acetazolamide (**AAZ**), clorgyline (CLO), and selegiline (SEL) are reported as reference drugs. nt: not tested. (nd: not detected; nt: not tested).

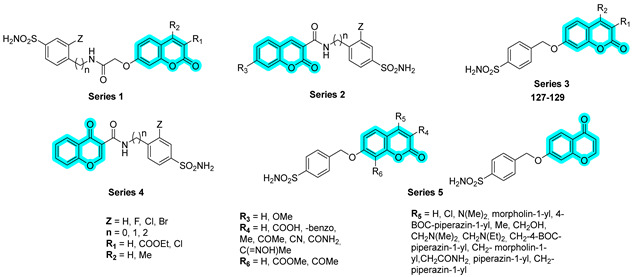												
Cmp	R_1_	R_2_	K_I_ (nM)							IC_50_ (nM)		
			hCA I	hCA II	hCA IV	hCA VA	hCA VB	hCA VII	hCA XII	hMAO-A	hMAO-B	SI
Serie 1	-	-	248–2400	1–161	49–421	7–133	14–107	8–167	9–106	nd	1700–10,850	1–6
Serie 2	-	-	575–3900	0.4–263	75–561	7–156	14–166	11–158	7–93	nd	15–324	30–673
**127**	H	H	544.3	6.1	46.7	37.1	15.5	8.2	37.8	nd	9.1	>1094
**128**	COOEt	H	765.7	20.8	39.5	89.9	83.5	9.7	16.5	nd	209.0	>47.8
**129**	Cl	Me	816.3	9.7	61.2	8.5	66.1	5.6	40.7	nd	6.7	>1502
Serie 4	-	-	228–2817	8–92	54–447	22–106	30–96	50–81	6–91	nd	33–670	8–274
Serie 5	-	-	533–88,000	0.1–155	93–541	7–294	8–230	8–212	1–321	921-nd	7-nd	1–1432
**MTZ**	-	-	50.0	14.0	6200	65.0	62.0	2.1	31.3	nt	nt	-
**AAZ**	-	-	250.0	12.0	74.0	63.0	54.0	2.5	5.7	nt	nt	-
**SEL**	-	-	-	-	-	-	-	-	-	15,610	47.6	328
**CLO**	-	-	-	-	-	-	-	-	-	3.0	2501	0.001

**Table 21:** In vitro K_I_ values of compounds **130**–**144** against hCA I, II, IX, and XII and IC_50_ values for α-glucosidase. Acetazolamide (**AAZ**) and acarbose are reported as reference drugs.

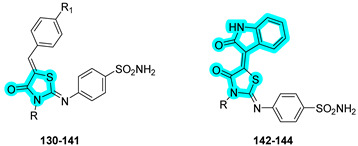							
Cmp	R	R_1_	K_I_ (nM)				IC_50_ (µM)
			hCA I	hCA II	hCA IX	hCA XII	α-Glucosidase
**130**	H	H	6434	90.5	72.4	119.8	0.6907
**131**	H	OCH_3_	2612	24.3	80.4	77.6	0.4750
**132**	H	Cl	8678	95.7	90.5	84.0	1.646
**133**	H	F	5415	7.0	64.1	19.9	0.4400
**134**	CH_3_	H	6345	44.5	61.5	74.5	0.3456
**135**	CH_3_	OCH_3_	4816	54.6	78.9	66.6	3.346
**136**	CH_3_	Cl	6331	29.1	59.5	48.8	1.378
**137**	CH_3_	F	4429	30.7	42.9	37.0	0.8597
**138**	CH_2_CH_3_	H	8747	77.2	52.4	96.3	2.025
**139**	CH_2_CH_3_	OCH_3_	6205	66.3	55.5	56.6	1.085
**140**	CH_2_CH_3_	Cl	6511	30.5	30.8	36.3	0.4470
**141**	CH_2_CH_3_	F	4416	85.0	33.9	11.0	1.276
**142**	H	-	630.8	23.0	47.0	33.5	1.833
**143**	CH_3_	-	95.5	40.7	40.1	841	0.5991
**144**	CH_2_CH_3_	-	675.5	105.4	20.1	9.1	0.9781
**AAZ**	-	-	250.0	12.0	25.0	5.7	-
**Acarbose**	-	-	-	-	-	-	0.4206

**Table 22:** Inhibition data for hCAs I and II and bacterial CAs CynT2 (*E. coli* β-CA), EcoCA (*E. coli* γ-CA), and NgCA (*N. gonorrhoeae* α-CA) with compounds **145**–**164** using the standard drug **AAZ** in a stopped-flow CO_2_ hydrase assay.

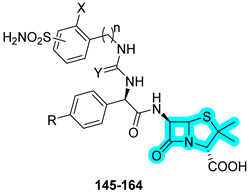										
Cmp	R	n		X	Y	K_I_ (nM)				
						hCA I (α)	hCA II (α)	CynT2 (β)	EcoCAγ (γ)	NgCAα (α)
**145**	H	0	3-SO_2_NH_2_	H	O	312.1	211.1	43.4	512.7	84.3
**146**	H	0	4-SO_2_NH_2_	H	O	309.7	36.1	76.0	220.8	22.2
**147**	H	0	4-SO_2_NH_2_	F	O	246.3	24.9	131.9	165.7	59.8
**148**	H	0	4-SO_2_NH_2_	Cl	O	562.1	241.8	189.1	82.1	65.4
**149**	H	0	4-SO_2_NH_2_	Br	O	344.3	56.0	218.2	97.3	169.6
**150**	H	1	4-SO_2_NH_2_	H	O	361.7	66.4	431.7	186.2	92.3
**151**	H	2	4-SO_2_NH_2_	H	O	291.9	43.8	445.7	78.9	71.8
**152**	H	0	3-SO_2_NH_2_	H	S	598.1	215.3	94.6	504.5	101.3
**153**	H	0	4-SO_2_NH_2_	H	S	384.3	65.6	123.4	199.2	46.2
**154**	H	0	4-SO_2_NH_2_	Cl	S	601.5	347.3	214.3	121.7	83.8
**155**	H	0	4-SO_2_NH_2_	Br	S	355.0	71.0	266.9	170.3	291.2
**156**	H	1	4-SO_2_NH_2_	H	S	419.4	84.6	487.1	207.3	145.7
**157**	H	2	4-SO_2_NH_2_	H	S	259.7	29.2	461.0	98.3	69.2
**158**	OH	0	4-SO_2_NH_2_	H	O	369.6	65.1	71.3	216.2	7.1
**159**	OH	0	3-SO_2_NH_2_	H	S	614.6	263.8	89.7	515.8	37.5
**160**	OH	0	4-SO_2_NH_2_	H	S	412.8	73.7	114.2	162.0	78.1
**161**	OH	0	4-SO_2_NH_2_	Cl	S	617.2	434.1	191.4	132.4	59.7
**162**	OH	0	4-SO_2_NH_2_	Br	S	373.4	108.8	228.7	161.5	201.5
**163**	OH	1	4-SO_2_NH_2_	H	S	442.3	101.4	463.8	184.8	113.6
**164**	OH	2	4-SO_2_NH_2_	H	S	303.9	47.2	428.2	83.3	41.2
**AAZ**	-	-	-	-	-	250	12.5	227	248	74.1

**Table 23:** Inhibition data for hCAs I, II, IX, and XII with compounds **165**–**167** using the standard drug **AAZ** in a stopped-flow CO_2_ hydrase assay.

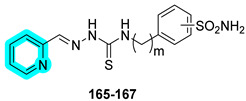								
Cmp	m		K_I_ (nM)				SI I/IX // II/IX	SI I/XII // II/XII
			hCA I	hCA II	hCA IX	hCA XII		
**165**	0	4-SO_2_NH_2_	207	156.6	351.9	4.9	0.59 // 0.45	42.2 // 31.9
**166**	0	3-SO_2_NH_2_	538	2.5	4.9	5.6	109.8 // 0.49	96.1 // 0.43
**167**	2	4-SO_2_NH_2_	270	83.0	28.5	9.3	9.5 // 2.9	29.0 // 9.0
**AAZ**	-	-	250	12.0	25.0	5.7	10.0 // 0.48	43.9 // 2.1

**Table 24:** In vitro K_I_ values of compounds **168**–**183** against hCA I, II, IX, and XII and IC_50_ values for cyclin-dependent kinases 4 and 6. Palbociclib (Pal) and acetazolamide (**AAZ**) are reported as reference drugs.

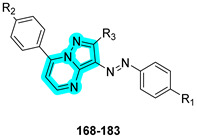									
Cmp	R_1_	R_2_	R_3_	K_I_ (nM)				IC_50_ (µM)	
				hCA I	hCA II	hCA IX	hCA XII	CDK4	CDK6
**168**	SO_2_NH_2_	H	OH	618	84.3	64.2	71.4	-	-
**169**	SO_2_NH_2_	CH_3_	OH	782	90.4	66.9	74.5	-	-
**170**	SO_2_NH_2_	OCH_3_	OH	621	57.3	40.6	20.2	0.672	0.111
**171**	SO_2_NH_2_	F	OH	439	21.8	11.2	41.8	0.292	0.054
**172**	SO_2_NH_2_	Cl	OH	814	30.6	24.6	48.9	-	-
**173**	SO_2_NH_2_	Br	OH	1260	58.9	35.1	44.2	-	-
**174**	COOH	H	OH	14,200	6152	2148	1537	-	-
**175**	COOH	OCH_3_	OH	11,300	4482	2744	849	-	-
**176**	COOH	F	OH	8961	3127	926	1294	1.274	0.422
**177**	COOH	Br	OH	10,700	3592	1261	2011	-	-
**178**	SO_2_NH_2_	H	CH_3_	895	60.0	25.6	14.8	-	-
**179**	SO_2_NH_2_	CH_3_	CH_3_	1142	51.5	18.4	30.5	-	-
**180**	SO_2_NH_2_	OCH_3_	CH_3_	2648	44.8	31.6	8.7	-	-
**181**	SO_2_NH_2_	F	CH_3_	663	76.1	19.7	26.1	0.723	0.069
**182**	SO_2_NH_2_	Cl	CH_3_	927	68.2	46.8	66.4	-	-
**183**	SO_2_NH_2_	Br	CH_3_	3418	33.2	51.9	59.2	-	-
**PAL**	-	-	-	-	-	-	-	0.072	0.046
**AAZ**	-	-	-	250	12.0	25.0	5.7	-	-

## Data Availability

Not applicable.
